# Prolonged Blood-Brain Barrier Injury Occurs After Experimental Intracerebral Hemorrhage and Is Not Acutely Associated with Additional Bleeding

**DOI:** 10.1007/s12975-018-0636-9

**Published:** 2018-06-14

**Authors:** Colby A. Nadeau, Kristen Dietrich, Cassandra M. Wilkinson, Andrew M. Crawford, Graham N. George, Helen K. Nichol, Frederick Colbourne

**Affiliations:** 1 Department of Psychology, University of Alberta, P217 Biological Sciences Building, Edmonton, Alberta T6G 2E9 Canada; 2 Neuroscience and Mental Health Institute, University of Alberta, Edmonton, Canada; 3 Molecular and Environmental Sciences Group, Department of Geological Sciences, University of Saskatchewan, Saskatoon, Canada; 4 Department of Chemistry, University of Saskatchewan, Saskatoon, Canada; 5 Department of Anatomy and Cell Biology, University of Saskatchewan, Saskatoon, Canada

**Keywords:** Blood-brain barrier, Gadolinium extravasation, Ion dyshomeostasis, Intracerebral hemorrhage, X-ray fluorescence imaging

## Abstract

Intracerebral hemorrhage (ICH) causes blood-brain barrier (BBB) damage along with altered element levels in the brain. BBB permeability was quantified at 3, 7, and 14 days with Evans Blue dye after collagenase-induced ICH in rat. At peak permeability (day 3), a gadolinium (Gd)-based contrast agent was injected to further characterize BBB disruption, and X-ray fluorescence imaging (XFI) was used to map Gd, Fe, Cl, and other elements. XFI revealed that Ca, Cl, Gd, and Fe concentrations were significantly elevated, whereas K was significantly decreased. Therefore, using Gd-XFI, we co-determined BBB dysfunction with alterations in the metallome, including those that contribute to cell death and functional outcome. Warfarin was administered 3 days post-ICH to investigate whether additional or new bleeding occurs during peak BBB dysfunction, and hematoma volume was assessed on day 4. Warfarin administration prolonged bleeding time after a peripheral cut-induced bleed, but warfarin did not worsen hematoma volume. Accordingly, extensive BBB leakage occurred after ICH, but did not appear to affect total hematoma size.

## Introduction

Intracerebral hemorrhage (ICH), characterized by one or more vessels rupturing in the parenchyma, has a > 40% mortality rate. Currently, ICH accounts for 10–15% of all strokes and often leads to lifelong disabilities in its survivors [1]. Anticoagulants such as warfarin contribute to ~ 20% of hemorrhage cases and may transform asymptomatic microhemorrhages (cerebral microbleeds; CMBs) into symptomatic ICH [2, 3]. Newer anticoagulants do not increase the risk of ICH but negatively impact outcome if ICH occurs [4].

Multiphasic blood-brain barrier (BBB) dysfunction is observed post-ICH. Acute BBB dysfunction occurs at ictus, and delayed BBB opening is related to secondary injury (e.g., inflammation) and repair (e.g., angiogenesis) processes [5, 6]. Increased BBB permeability in the region surrounding the hematoma (peri-hematoma zone; PHZ) is caused by extravasated blood components and secondary injury processes (for review, see [7]). Indeed, intraparenchymal injections of blood components, such as lysed erythrocytes or iron, induce BBB dysfunction along with edema, cell death, and behavioral dysfunction [8, 9]. Acute increases in matrix metalloproteinase (MMP) levels also contribute to BBB dysfunction and may worsen bleeding or cause additional bleeding by weakening blood vessels and increasing the likelihood of rupture [10–13]. The type IV collagenase used to induce ICH is MMP-9, so it is possible that delayed bleeding occurs in the collagenase model of ICH. Finally, angiogenesis begins 1 week after ICH and causes delayed BBB permeability [14, 15].

Clinically, BBB permeability is assessed with magnetic resonance imaging of gadolinium (Gd)-based contrast agents [16]. In patients, there is high PHZ BBB permeability 1-week post-ICH [17]. Similarly, in the collagenase animal model, BBB permeability is persistently elevated from 5 h to 7 days after stroke, with function restored by day 14 [18, 19]. Persistent BBB damage is concerning as the free passage of ions and molecules into the parenchyma is potentially harmful [20]. Changes in BBB permeability, edema, and ion dyshomeostasis are causally related. Ion dyshomeostasis (e.g., alterations in Fe, K, Cl, and Na levels) occurs in both the autologous whole blood model (AWB) and the collagenase model of ICH, albeit with different temporal progressions. Dyshomeostasis occurs within 24 h of ictus in AWB [21]. In the collagenase model, alterations in Fe occur within 1–3 days of ICH, with changes in Cl, Fe, and K noted at day 14 in the collagenase model [22, 23]. Acutely, edema contributes to alterations in K, Cl, and Na, although K and Cl (and likely Na) dyshomeostasis persists long after edema is thought to be resolved (i.e., after days 3–7 in animal models) [24]. Edema and BBB dysfunction are directly related, as large molecule (e.g., albumin) extravasation contributes to vasogenic edema [25].

Ion dyshomeostasis can be measured and spatially assessed using X-ray fluorescence imaging (XFI) [26]. This technique is preferred over traditional assays performed on extracted tissue pieces because XFI allows for fine spatial resolution and a clear delineation between the hematoma and PHZ [27], which is a key area of ongoing tissue injury and repair. Inductively coupled plasma mass spectrometry (ICP-MS) is another sensitive technique for measuring ion levels that relies on accurate dissection of tissue of interest [28]. However, ICP-MS can suffer from incomplete ionization, variability in ionization efficiency for different elements (the so-called matrix effects), and lack of spatial resolution. In comparison, XFI can measure total element content accurately after ICH as it is always detectable without chemical pre-treatment, has minimal matrix effects, and readily detects both bound and free metal ions for most biologically relevant metals [27].

Here, BBB injury after ICH was assessed with methodology combining XFI and Gd-based contrast agents after we measured the time course of BBB permeability using an Evans Blue assay. At peak BBB permeability (day 3 post-ICH), we measured Gd concentration as a separate measure of BBB dysfunction with XFI. This method allowed for detailed spatial analysis of BBB damage, while allowing us to directly relate BBB permeability to PHZ ion dyshomeostasis. We are the first to use this approach in rodent stroke models, although others have measured Gd with synchrotron techniques to assess other pathologies [29, 30]. XFI was compared to ICP-MS. Finally, we use traditional blood volume measurement methods after giving an oral anticoagulant to increase the size of any bleeding (e.g., hematoma expansion or new bleeds) that might exist 3 days post-ICH, a time of high BBB dysfunction.

## Materials and Methods

### Subjects

One hundred four male Sprague Dawley rats (350–450 g) were obtained from Charles River (Saint Constant, QC). Rats were individually housed in a temperature- and humidity-controlled room with a 12-h light cycle with food and water provided ad libitum.

## Experiment 1: Time Course of BBB Permeability After ICH

### Experimental Groups

Animals were randomly assigned to either the control surgery (SHAM) or ICH groups (*n* = 5 SHAM; *n* = 36 collagenase). There is low variability in EB concentration in SHAM animals; thus, fewer rats were needed in this group [31]. Collagenase animals were randomly assigned to three-day (3D; *n* = 13), seven-day (7D; *n* = 13), or 14-day (14D; *n* = 10) survival groups.

### Intracerebral Hemorrhage

A collagenase model of ICH was used [18]. Briefly, rats were anesthetized using isoflurane (4% induction; 2% maintenance; 60% N_2_O, remainder O_2_). A midline incision was made, and a hole was drilled 0.5 mm anterior and 3.5 mm left of bregma. A 26-gauge Hamilton syringe (Hamilton, Reno, NV, USA) was lowered 6.5 mm into the striatum, and 0.14 U of bacterial collagenase (type IV-S; Sigma, Oakville, ON, Canada) was injected over 5 min. The needle was withdrawn after 5 min, a screw sealed the hole, and the incision was closed. Marcaine was used as an analgesic. Body temperature was maintained at 37 °C using a rectal temperature probe and water blanket. SHAM animals underwent similar procedures, but after the midline incision was made, they were simply kept anesthetized for the same duration as a collagenase surgery, and then the incision was closed. The skull was not opened to keep the BBB intact.

### Evans Blue Spectroscopic Assay

EB extravasation was assessed as done previously [31]. Briefly, EB dye (Sigma; 2% in saline; 4 mg/kg; filter sterilized) was injected into the tail vein. Dye circulated 2 h before rats were anesthetized and then transcardially perfused with saline. Each hemisphere (ipsilateral, IPSI; contralateral, CONTRA) was homogenized in 0.1 M PBS. The sample was centrifuged, and the supernatant was incubated at 4 °C with trichloroacetic acid (50% *w*/*v*; Fisher Scientific, Whitby, ON) and centrifuged. Absorbance was read at 610 nm. EB extravasation was determined based on a standard curve.

## Experiment 2: Acute Ion Dyshomeostasis After ICH

### Experimental Groups

Rats underwent collagenase (*n* = 5) or SHAM (*n* = 5) surgeries as described in experiment 1 and euthanized 72 h later for XFI and ICP-MS analysis.

### Magnevist® Injections

Magnevist® (gadopentetate dimeglumine; 2.5 mL/kg; Bayer, Mississauga, ON), which has a short half-life, was injected into the tail vein and allowed to circulate for 10 min [32]. Rats were decapitated. Brains were removed within 90 s and rapidly frozen in cooled isopentane. The tissue was not perfused as that disrupts element concentrations and introduces confounds. Brain tissue was cryostat sectioned at the largest hematoma cross-section for XFI imaging.

### Inductively Coupled Plasma Mass Spectrometry

One-millimeter-thick sections for ICP-MS analysis were taken from flash frozen tissue (at maximum hematoma) following cryostat sectioning for XFI (below). Ipsilateral sections were dissected into hematoma and PHZ and contralateral sections were dissected to include the striatum. Tissue regions were digested in high purity nitric acid for 1 week. Ca, Fe, Gd, K, and Na concentrations were measured using ICP-MS (Thermo Scientific ICAP-Q quadrupole ICP-MS, Canadian Centre for Isotopic Microanalysis, University of Alberta). Cl concentrations could not be determined with this method.

### X-ray Fluorescence Imaging

Tissue sections (thickness, 20 μM) were mounted on metal-free Thermanox coverslips (Thermo Scientific, Waltham, MA). Data were collected at the Stanford Synchrotron Radiation Lightsource (SSRL) at beamline 10-2 as described previously [22]. Briefly, maps of Ca, Cl, Fe, K, and Gd were collected using a 35-μm aperture and an incident X-ray energy of 13,450 eV. Due to the overlap of the Gd Lβ1 emission (6.7 keV) with Fe Kα emissions (6.4 keV), a few images were rescanned at an incident beam energy of 7700 eV (below the Gd L2 edge) and both the Fe and Gd images from both scans were compared. There was no quantitative difference between Fe at 7700 and 13,450 eV. Samples were continuously scanned with an effective dwell time of 200 ms per 30-μm step and X-ray fluorescence was detected with a single-element Vortex silicon drift detector (Hitachi High Tech. USA, Chatsworth, CA) and read out with Xspress 3 (Quantum Detectors, Chilton Oxfordshire, UK) digital signal processing electronics.

X-ray fluorescence emission spectra were fit using the M-BLANK program [33, 34]. A blank spectrum was calculated using data from pixels at which no tissue was present and was then subtracted from the data. The blank-subtracted corrected x-ray fluorescence spectra for each pixel were then fit in a linear least square sense using a series of modified Gaussians with the energy calibration, Gaussian widths, and elemental branching ratios fixed at the experimentally determined reference values [35, 36]. Fluorescence intensities were converted to aerial concentrations (μg/cm^2^) using reference standards deposited on 6.3-μm-thick Mylar film: K and Cl (KCl, 98.8 μg/cm^2^), Ca (CaF2, 56.8 μg/cm^2^), Gd (GdF3, 53.7 μg/cm^2^), and Fe (Fe 56.0 μg/cm^2^) (Micromatter, Vancouver, CA).

Data were reformatted with custom software for use in ImageJ (version 1.50b, National Institutes of Health, Bethesda, MD). Elemental concentrations were determined for CONTRA striatum, IPSI hematoma center (HEM center), IPSI hematoma edge (HEM edge), and SHAM striatum. Since there was no significant difference between SHAM and CONTRA striatum (*t* test; *p* > 0.05), HEM center and HEM edge were compared to CONTRA striatum only. Based upon previous work, the hematoma border was defined as a sharp change in Fe, indicating a transition from blood (hematoma) to tissue (PHZ) in ImageJ [22]. The hematoma border was applied to all element channels per sample. Three regions of interest (ROIs) were taken for distance analyses for Ca, Gd, Fe, Cl, and K and were performed from the hematoma edge to 1260 μm. All ROIs were averaged together in 180 × 180 μm bins.

## Experiment 3: Testing for Late Bleeding After ICH Onset

### Experimental Groups

Animals were randomized into warfarin (*n* = 24) or vehicle control (*n* = 24) prior to surgery. With our sample size, we had 80% power to detect a 25% difference in hematoma volume.

### Warfarin Preparation and Administration

Warfarin (Sigma-Aldrich, PHR1435-1G) was dissolved in distilled water at 3.5 mg/kg. A 0.5 mg/kg of warfarin (experimental group) or equivalent volume of dH_2_O (control) was mixed into small samples of cookie dough (Pillsbury) to administer the drug orally 72 h post-ICH [37]. Complete ingestion of dough was visually confirmed. As warfarin has a half-life between 20 and 60 h, we expected that warfarin had a pharmacologically active anticoagulant effect starting several hours after dosing (initially with decreasing factor VII levels) until euthanasia at 24 h later. Route of administration was selected to minimize stress and negate the need for anesthetics, both of which could influence BBB integrity [38, 39]. A non-lethal dosage was selected to produce a clinically relevant increase in bleeding time (i.e., international normalized ratio 2–3× longer than normal).

### Tail Bleed Assessment

To verify that the selected warfarin dose was physiologically active, a tail bleed assessment was done immediately prior to euthanasia (modified from [37]). Animals were anesthetized with isoflurane. Tails were severed 2 mm from tip. Bleed time was assessed by placing filter paper to the incision and replacing the paper every 10 s. The bleed was deemed clotted when two consecutive papers displayed no blood. Cerebral hematoma volume was assessed immediately afterwards.

### Hemoglobin Assay

Cerebral blood volume was assessed as previously described [22]. Briefly, rats were euthanized 24 h after vehicle or warfarin administration, with the latter given to aggravate bleeding from the initial bleeds or from new CMBs. Brains were extracted, and a spectrophotometric hemoglobin assay was performed to measure blood volume. Hematoma volume was calculated as IPSI blood volume − CONTRA blood volume.

## Statistical Analysis

Data were analyzed using GraphPad Prism (v. 6.0, GraphPad Software Inc., La Jolla, CA). Two-way ANOVAs were used in experiment 1 to assess time × hemisphere effects. One-way ANOVAs were used in experiment 2 to assess region effects. Repeated measures ANOVA was used to assess distance main effects. For experiment 3, hematoma volume was assessed with ANOVA. Tail bleed was evaluated with Student’s *t* test with Welch’s correction to adjust for unequal variances. Bonferroni’s post hoc tests were used in experiments 1, 2, and 3. Bartlett’s tests were used to assess assumptions of equal variance. If variance was not equal, the Kruskal-Wallis tests were used. Proportions in experiment 1 were compared with a chi-squared test. Statistical significance was defined as *p* < 0.05. All data are presented as mean ± standard deviation (SD).

## Results

### Mortality and Exclusions

There was no mortality. In experiment 1, one SHAM sample was excluded from analysis due to experimenter error at the time of euthanasia. In experiment 2, two SHAM samples were not imaged due to time constraints at SSRL. In experiment 3, one vehicle control animal was excluded due to experimenter error.

## Experiment 1

### Blood-Brain Barrier Permeability Peaks Early and Decreases Thereafter

**Fig. 1:**
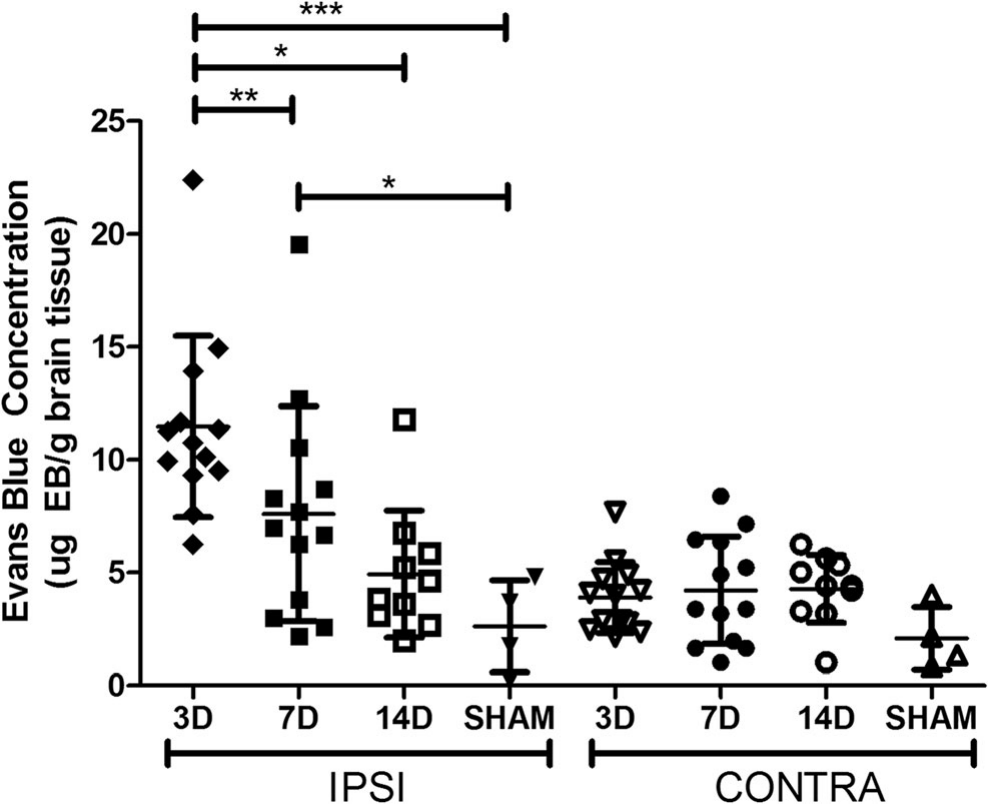
Evans Blue extravasation at 3D was significantly higher than at 7D and 14D in IPSI tissue. Ipsilateral EB extravasation at 3D was significantly higher than SHAM. Significant elevations persisted to 7D. There were no differences in EB extravasation in the CONTRA hemisphere. There was a significant relationship between time and proportion of samples with BBB dysfunction in the IPSI, but not CONTRA, hemisphere (mean ± SD; *p < 0.05; **p < 0.01; ***p < 0.001)

There was a main effect of day (*p* < 0.001) and hemisphere (*p* < 0.001) with greater EB extravasation in the IPSI hemisphere compared to CONTRA. Ipsilateral 3D BBB permeability was significantly higher than 7D (*p* < 0.01), 14D (*p* < 0.001), and SHAM (Fig. 1; *p* < 0.001). 7D IPSI BBB permeability was higher than SHAM (*p* < 0.05). There were no differences in BBB permeability among CONTRA samples (*p* = 0.117).

### A Subset of Animals Display BBB Dysfunction

High variability was noted (Fig. 1) and so BBB dysfunction was categorized as any sample with EB extravasation above that of the highest SHAM level. This was observed in the IPSI hemisphere in 100, 69.2, and 40% of animals at 3D, 7D, and 14D, respectively. In CONTRA samples, 23.1, 46.2, and 40% displayed dysfunction at days 3, 7, and 14, respectively. A chi-square test revealed a significant relationship between day and BBB permeability in the IPSI (*p* < 0.01), but not CONTRA hemisphere (Fig. 1; *p* = 0.451).

## Experiment 2

### Ion Dyshomeostasis and BBB Disruption Following ICH Can Be Detected with ICP-MS

**Fig. 2:**
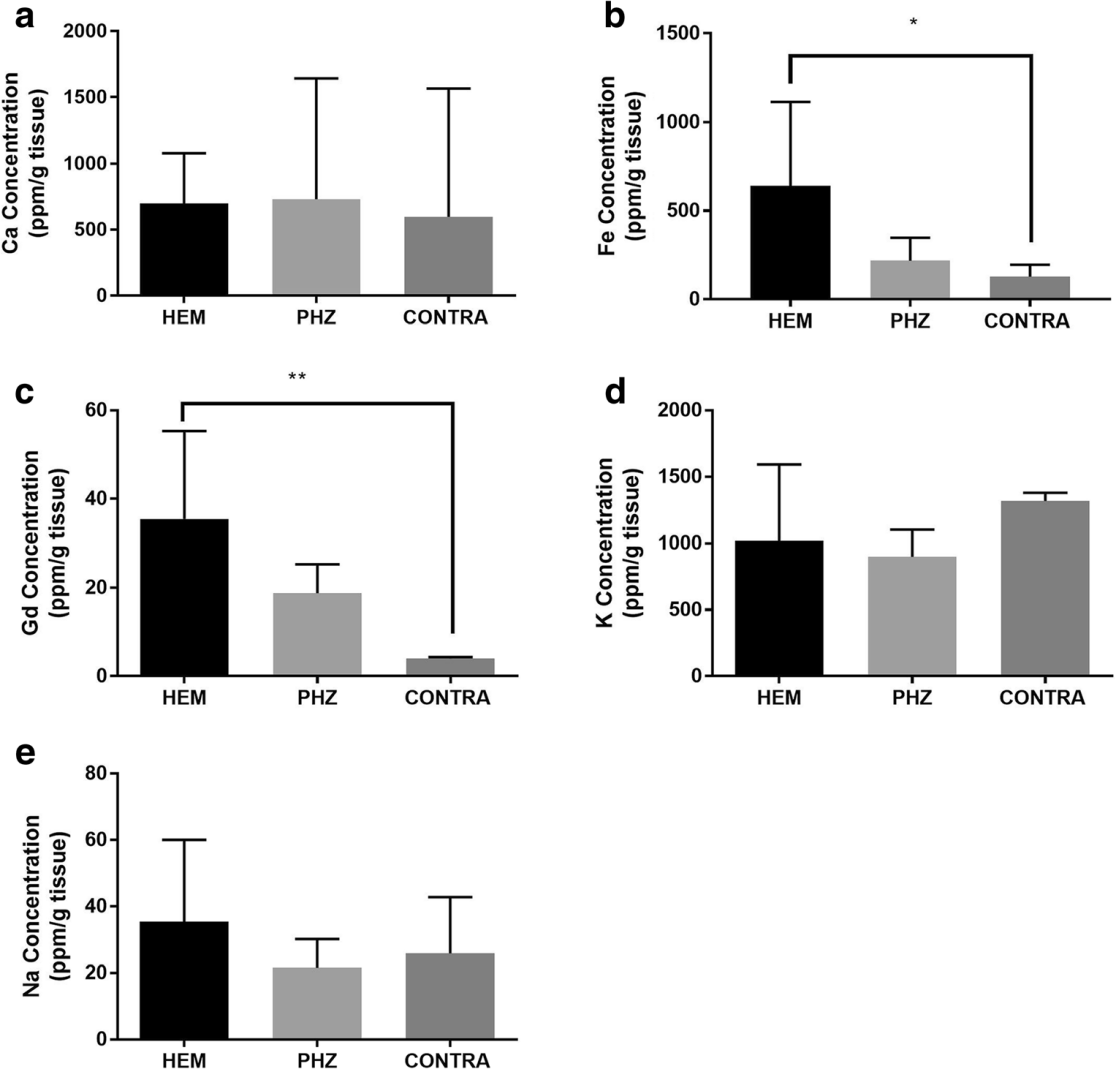
ICP-MS measurement of element concentrations after ICH. Concentrations of Ca (**a**) within the HEM did not differ from CONTRA tissue. Fe (**b**) and Gd (**c**) concentrations were significantly increased in the HEM as compared to CONTRA striatum. Concentrations of K (**d**) and Na (**e**) within the HEM did not differ from CONTRA tissue (mean ± SD; *p < 0.05; **p < 0.01; ***p < 0.001)

Ca, K, and Na concentrations did not differ among the HEM, PHZ, and CONTRA striatum as measured by ICP-MS (Fig. 2a, d, e; *p* > 0.05). ICP-MS analysis revealed that Gd could be detected in brain tissue when a dose of 2.5 mL/kg of Magnevist was injected in the tail vein and allowed to circulate for 10 min (Fig. 2c). HEM Fe and Gd concentrations were significantly higher than CONTRA values (Fig. 2b, c; *p* = 0.040, *p* = 0.003, respectively).

### Intracerebral Hemorrhage Induces Element Alterations in the HEM and PHZ as Measured with XFI

**Fig. 3:**
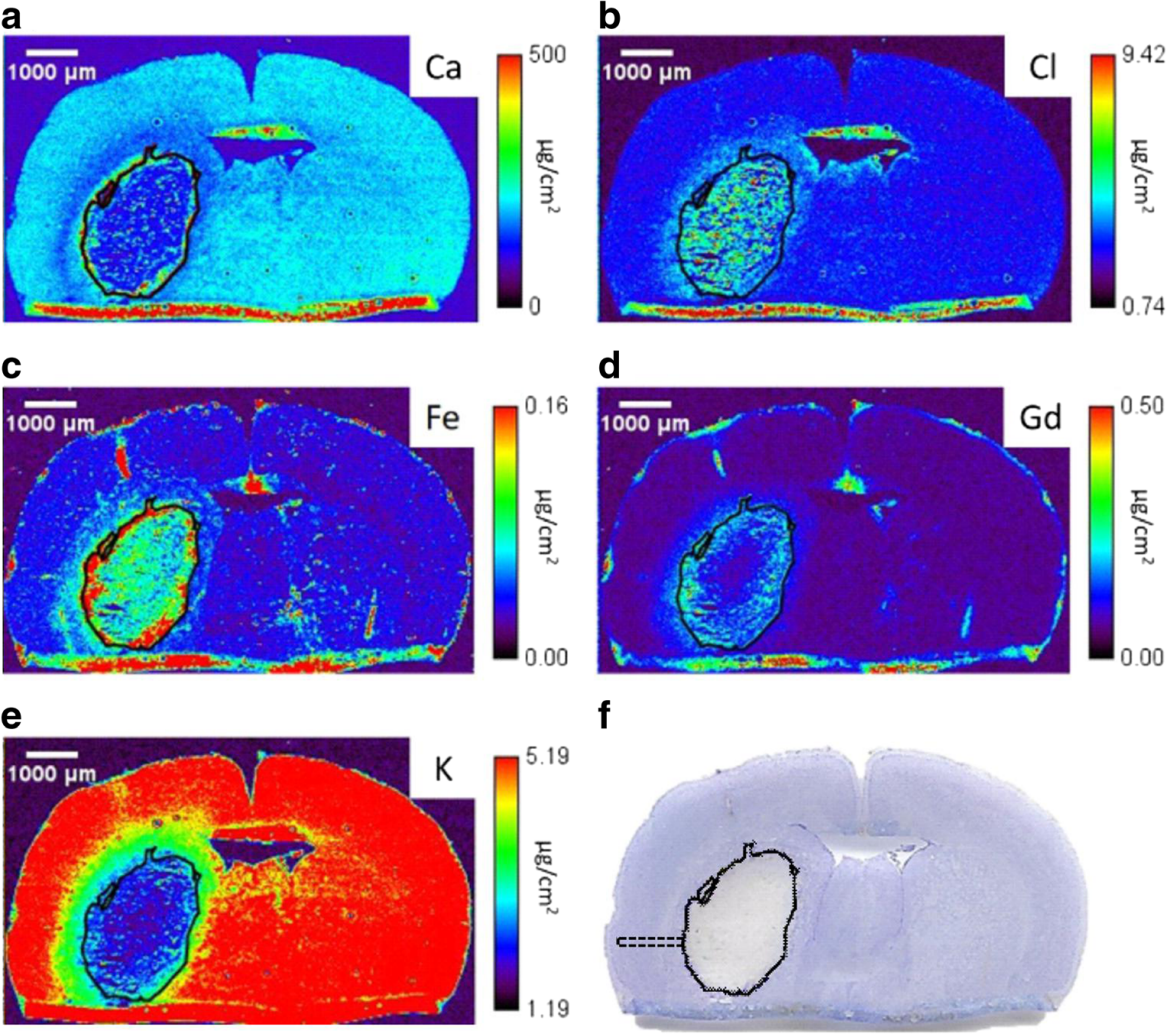
Representative x-ray fluorescent images of Ca (**a**) Cl (**b**), Fe (**c**), Gd (**d**), and K (**e**). The solid black line marks the HEM boundary, which was determined in the Fe channel by locating a sharp decrease in Fe levels. The HEM boundary was propagated to all other channels. Cresyl violet staining (**f**) confirms the HEM boundaries determined with XFI. A dotted black line illustrates a sample distance ROI (**f**). Three separate arrays of data collection were used to quantify PHZ element levels. The tissue fold at the bottom is an artifact from sectioning

**Fig. 4:**
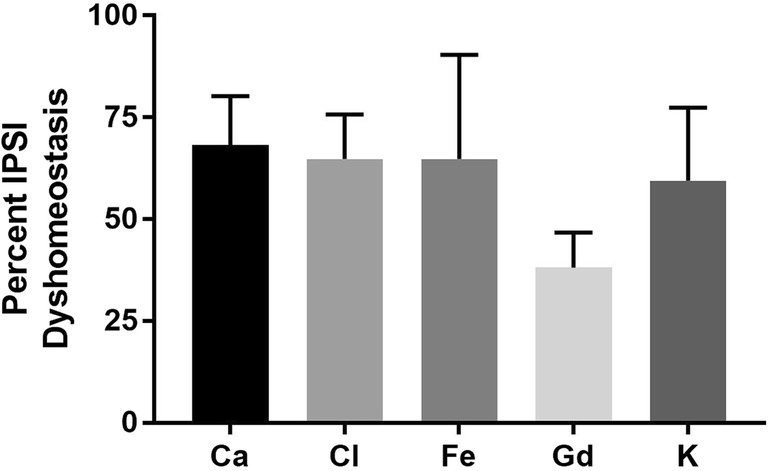
Percent of sampled tissue with greater than 25% change (arbitrary threshold likely to denote biological significance) in ion concentration as compared to within-subjects CONTRA striatum. Over 50% of IPSI tissue sampled outside of the HEM showed Ca, Cl, Fe, and K dyshomeostasis, while ~ 40% of tissue showed Gd dyshomeostasis. Figure 3 shows representative x-ray fluorescent images

All visible hemorrhaging occurred in the striatum (Fig. 3). At the level containing the maximum hematoma area there was little Gd in the center of the HEM, with high Gd concentrations near the HEM edge (Fig. 3d). High Fe concentration presented inside the HEM boundary (Fig. 3c). Both Cl (Fig. 3b) and K (Fig. 3e) concentrations appeared relatively constant in the HEM, although a ring of slightly higher K concentration was observed inside the HEM edge. Data from all IPSI hemispheres were analyzed to determine the percent of tissue with ion dyshomeostasis (defined as > 25% change in ion levels) (Fig. 4). ~ 60% of tissue sampled showed Ca, Cl, Fe, and K dyshomeostasis, while ~ 40% of tissue showed Gd dyshomeostasis (Fig. 4).

**Fig. 5:**
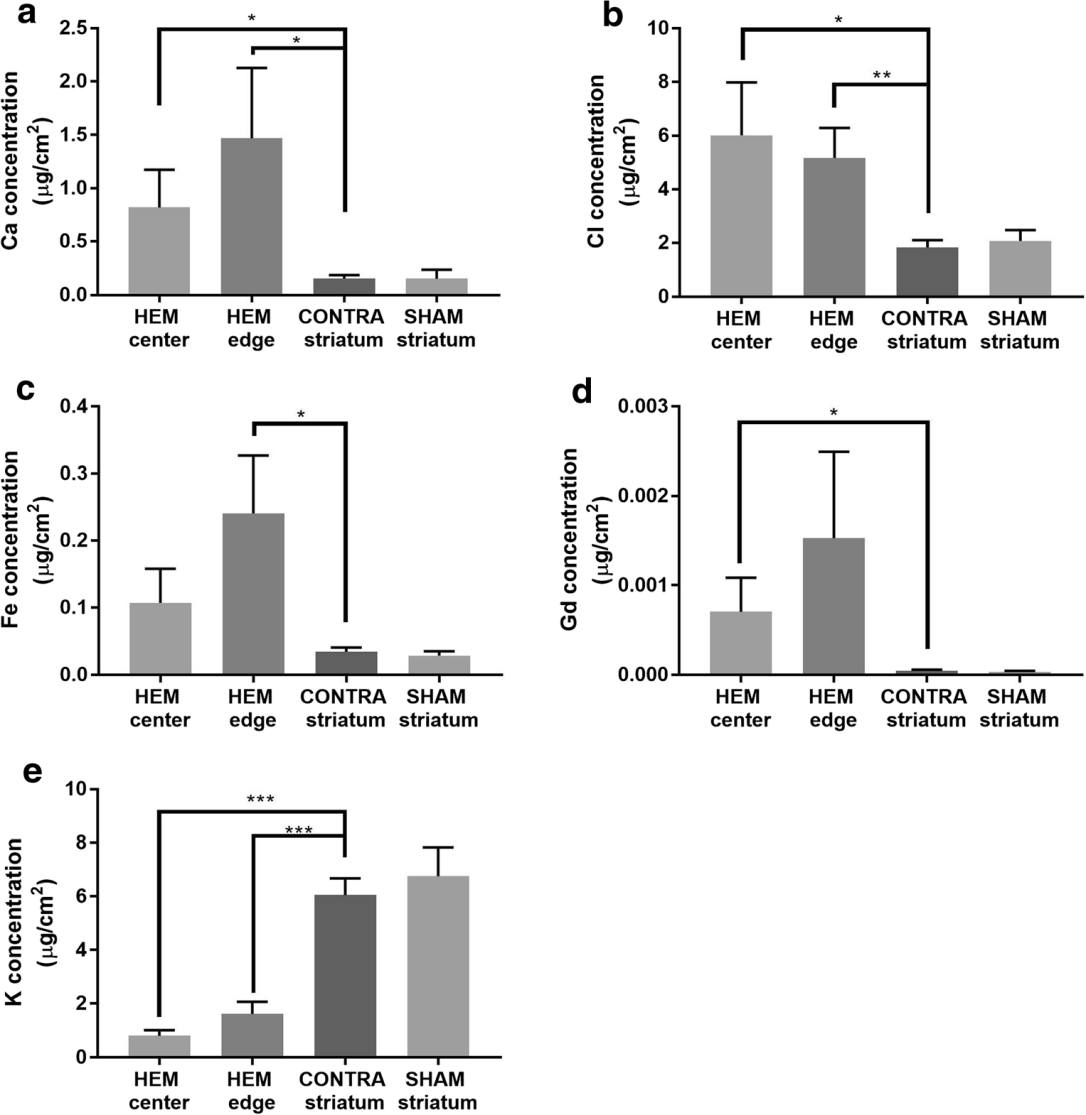
XFI measurement of element concentrations after ICH. Ca (**a**) and Cl (**b**) concentrations were significantly increased in the HEM center compared to CONTRA striatum. Fe (**c**) concentrations were significantly increased in the HEM edge compared to CONTRA striatum. Gd (**d**) concentrations were significantly increased in the HEM center compared to CONTRA striatum. K (**e**) concentrations were significantly decreased in the HEM center and edge compared to CONTRA striatum (mean ± SD; *p < 0.05; **p < 0.01; ***p < 0.001)

Concentrations of Ca and Cl were significantly increased in the HEM center (*p* = 0.027, *p* = 0.018, respectively) and HEM edge (*p* = 0.024, *p* = 0.004, respectively) as compared to CONTRA striatum (Fig. 5a, b). Fe concentration in the HEM center was significantly higher than the CONTRA striatum (Fig. 5c; *p* = 0.012), while Gd concentration at the HEM edge was significantly higher than CONTRA striatum (Fig. 5d; *p* = 0.043). K concentrations at HEM center (*p* < 0.001) and HEM edge (*p* < 0.001) were significantly lower than levels in CONTRA striatum (Fig. 5e).

**Fig. 6:**
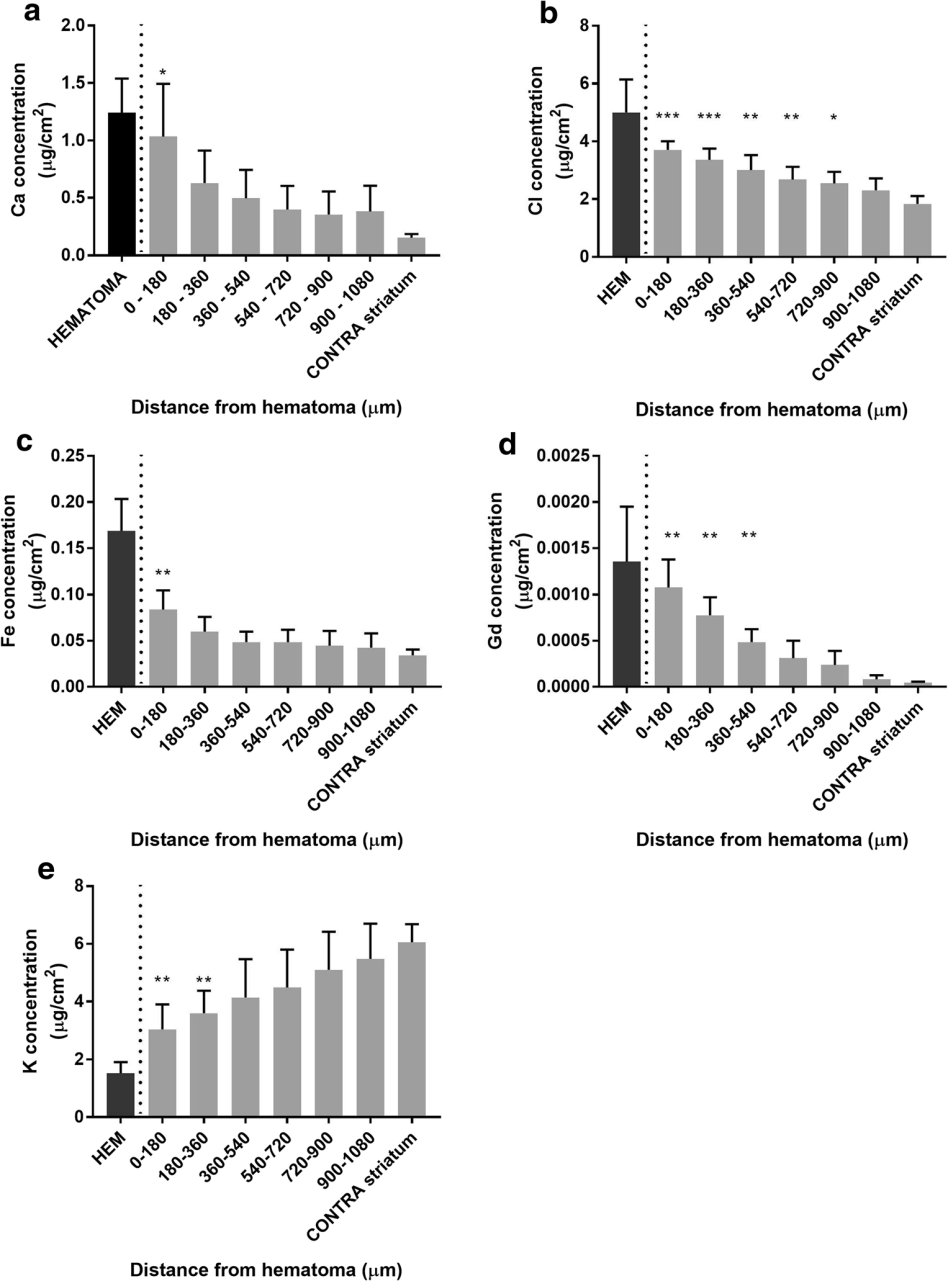
There was a distance main effect for Ca, Cl, Fe, and Gd, such that values were highest closest to the HEM and decreased with distance into the PHZ at 3D (**a**–**d**). There was a distance main effect for K, where values were lowest in the HEM and normalized with distance into the PHZ (**e**). See Fig. 3f for an illustration of sampling method (mean ± SD; *p < 0.05 as compared to CONTRA. ^‡^*p* < 0.05 as compared to SHAM)

Ca (Fig. 6a; *p* = 0.009, distance main effect), Cl (Fig. 6b; *p* < 0.001, distance main effect), Fe (Fig. 6c; *p* < 0.001, distance main effect), and Gd (Fig. 6d; *p* < 0.001, distance main effect) concentrations were highest in the HEM. Ca and Fe values differed from CONTRA values from 0 to 180 μm into the PHZ (Fig. 6a, c). Elevated Gd concentrations occurred from 0 to 540 μm into the PHZ (Fig. 6d), whereas Cl concentrations were significantly elevated from 0 to 900 μm into the PHZ (Fig. 6b). K was significantly lower than CONTRA values from 0 to 360 μm into the PHZ, respectively (Fig. 6e; *p* < 0.001, distance main effect).

## Experiment 3

**Fig. 7:**
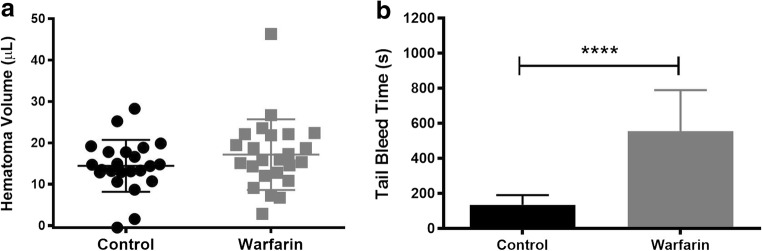
Warfarin did not significantly increase the cerebral HEM volume (**a**) although it did significantly increase the time it took peripheral blood to clot (**b**) (mean ± SD; *p < 0.05; **p < 0.01; ***p < 0.001)

Although warfarin did not significantly increase HEM volume (Fig. 7a; *p* = 0.1418), it tripled the time for the tail bleed to clot (Fig. 7b; *p* < 0.001).

### Discussion

Blood-brain barrier damage follows ICH and is related to secondary injury (e.g., edema, inflammation). Here, we present the novel use of XFI to measure BBB permeability using a Gd-based contrast agent. This method provides fine spatial resolution to measure BBB dysfunction and co-localize these changes with various elements that impact neural function and cell death. Using this method and a bulk assay, we confirm and expand upon the nature and progression of BBB injury in the collagenase model of ICH at various survival times. We are the first to investigate the magnitude of ion dyshomeostasis and BBB dysfunction in the PHZ 3 days post-ICH and to investigate whether late bleeding occurs during a period of high BBB permeability.

Ipsilateral BBB permeability peaked 3 days after ICH and decreased thereafter, with most leakiness resolved by 2 weeks post-hemorrhage. There was high variability in the resolution of BBB permeability, which could stem from ongoing injury (e.g., inflammation) or repair (e.g., angiogenesis) mechanisms. Furthermore, in 40% of animals at 2 weeks after ICH, both the ipsilateral and contralateral hemispheres had BBB dysfunction. BBB dysfunction measured by EB extravasation in the collagenase model in mice and in the thrombin model in rats is resolved 2 weeks after the insult; however, there is little data on late contralateral BBB dysfunction [40, 41]. Late IPSI BBB dysfunction may stem from angiogenesis in the PHZ. Indeed, delayed changes in the neurovascular unit, including the BBB, facilitate angiogenesis and have been observed between days 7 and 14 post-ICH in the ipsilateral hemisphere [42]. Alternatively, CONTRA BBB dysfunction may indicate inflammation, as an upregulation of pro-inflammatory cytokines endures to at least 7 days post-ICH [43]. Regardless, our warfarin data suggests that BBB dysfunction around day 3 does not result in additional bleeding. However, we cannot exclude the possibility that later (i.e., after 3 days) BBB injury can occasionally cause re-bleeding or lead to new CMBs or larger bleeds, which seems possible based upon our work with therapeutic hypothermia where late bleeding occurred [23].

Using XFI, we noted alterations in Ca, Cl, Fe, and K in the HEM after ICH. Due to time constraints at SSRL, we were underpowered to perform correlational analyses on this data. In accordance with our EB and ICP-MS data, as well as magnetic resonance imaging studies, we show elevated BBB permeability in the IPSI hemisphere acutely after ICH using XFI [17, 19, 44]. There was little Gd in the center of the HEM, which would have displaced normal vasculature. Further, any remaining brain tissue in that region would likely be ischemic, thereby preventing delivery of Gd to that area [44].

We found that the greatest ion dyshomeostasis in the PHZ occurred at the HEM/PHZ interface when measured using XFI. Like others, we found that ICP-MS and XFI analyses did not yield the same results [28]. We found that BBB dysfunction and the associated Fe and K ion dyshomeostasis likely affected any residual striatum, while Cl dyshomeostasis extended further into the PHZ. Furthermore, in ~ 60% of IPSI tissue surface area sampled, there was Ca, Cl, Fe, and K dyshomeostasis, while ~ 40% of tissue surface area had altered BBB permeability. As we did not image the entire section or multiple sections, these data do not represent the entire hemisphere. Nonetheless, these changes may have a significant impact, as cell death, ion dyshomeostasis, and other processes contribute to the ongoing behavioral dysfunctions observed acutely following experimental and clinical ICH [19, 45]. Gd content was elevated above background levels as far as 540 μm into the PHZ. Likely, this BBB dysfunction may contribute to persistent, diffuse ionic imbalances acutely after ICH, alongside or independently of ongoing injury (e.g., inflammation, edema) in the region.

In accordance with previous XFI and histochemical work, we confirm PHZ Fe accumulation at 72 h post-ICH [46, 23]. Interestingly, in all ICH animals, we observed low Fe levels in the center of the HEM, which was surrounded by a ring of concentrated Fe. As microglia/macrophages, which facilitate HEM resolution, localize at the HEM edge within 3 days of ICH and infiltrate the HEM with time, the pattern observed here may represent a summation of both Fe in the HEM itself and Fe internalized by microglia/macrophages (i.e., that present in hemoglobin) [47]. Conversely, the pressure exerted on the immediate PHZ might act to concentrate elements at the periphery of the bleed.

Early PHZ injury is seen after ICH and may both cause and result in changes in Cl and Na concentrations [48]. Like others, we demonstrate elevated Cl concentrations in the PHZ after ICH [22]. Alterations in the ratio of the chloride-cation cotransporters lead to Cl dyshomeostasis and cause a shift in GABA signaling polarity [49]. In ischemia, reversal of GABA polarity is initially neuroprotective, but later hampers recovery [50]. Future research should determine the nature of Cl dyshomeostasis after ICH and its impact on GABA signaling polarity. Injury in the PHZ may also be related to alterations in Na levels. We did not detect any changes in Na using ICP-MS, but as previously noted, ICP-MS can be unreliable and much depends upon isolating tissue samples (e.g., normal tissue in the sample dilutes alterations in Na). Increases in brain Na after ICH are associated with cytotoxic edema, increased bleeding, and increases in intracellular Cl [21, 51].

Potassium concentrations are locally decreased after ICH. Decreased K levels are associated with edema in ischemic stroke models, so these changes may be indicative of peri-hematomal and diffuse edema [52]. After ICH, astrocytes are activated in the PHZ within 72 h, and astrocyte elevations persist to day 14 [53, 54]. In line with our K data, astrocyte activation decreases from the HEM edge into the PHZ [55]. Future work should assess where the K dyshomeostasis occurs (e.g., in neurons, astrocytes, extra-, or intracellularly) as we were unable to discern this with our XFI protocol.

To our knowledge, we are the first to investigate the presence of very delayed bleeding in the collagenase model of ICH, and for this we used warfarin, an anti-coagulant, to aggravate and thereby increase detection of any source of delayed bleeding using the traditional blood volume measurement method. We expected that heightened BBB permeability would increase delayed bleeding. However, warfarin administered 3 days after post-ICH did not increase HEM volume despite significantly worsening peripheral clotting. This suggests that new CMBs and/or hematoma expansion does not normally occur around day 3 post-ICH in the collagenase model, and that the vascular damage will not spontaneously generate further bleeding. However, CMBs may have occurred and were of an undetectable volume. A higher dose of warfarin might have allowed us to detect such small bleeds but could have caused mortality. Second, late re-bleeds or new CMBs may occur later than 3 days after ICH, and thus more prolonged warfarin use may be needed to aid in their detection. High-resolution methods such as resonance Raman spectroscopy (as used in [22]) are recommended for future studies as the standard hemoglobin assay includes measurement of hemoglobin breakdown products (e.g., heme, bilirubin) (Colbourne lab, unpublished data). Finally, late bleeding may be a combined result of multiple factors in ICH models. For instance, previous work found that animals with BBB damage, coagulopathy, and blood pressure alterations exhibited increased HEM volume [23]. Instead, our current data suggests that the BBB damage and coagulopathy on day 3 without blood pressure alterations is not sufficient to aggravate the initial bleeding.

### Conclusions

The use of Gd-based contrast agents to assess BBB permeability has many applications in the experimental stroke field. Here, we present a novel, spatially sensitive technique to concurrently assess BBB permeability and ion dyshomeostasis using a Gd-based contrast agent and XFI. We advocate for the use of XFI over other methods due to its precise measurements and fine spatial resolution. Nonetheless, other tracers should be used to get a more complete picture of BBB integrity after ICH. Use of the methodology presented here will further our understanding of changes in brain function after ICH in the PHZ and more distal regions.
